# Combination Effect of Silver Nanoparticles and Histone Deacetylases Inhibitor in Human Alveolar Basal Epithelial Cells

**DOI:** 10.3390/molecules23082046

**Published:** 2018-08-15

**Authors:** Sangiliyandi Gurunathan, Min-hee Kang, Jin-Hoi Kim

**Affiliations:** Department of Stem Cell and Regenerative Biotechnology, Konkuk University, Seoul 05029, Korea; pocachippo@gmail.com

**Keywords:** silver nanoparticles, MS-275, combination therapy, cytotoxicity, apoptosis, oxidative stress, autophagosomes, DNA damage

## Abstract

Although many treatment strategies have been reported for lung disease, the mechanism of combination therapy using silver nanoparticles (AgNPs) and histone deacetylases inhibitors (HDACi) remains unclear. Therefore, innovative treatment strategies are essential for addressing the therapeutic challenges of this highly aggressive lung cancer. AgNPs and HDACi seem to be the best candidates for anticancer therapy because of their anti-proliferative effect in a variety of cancer cells. First, we synthesized AgNPs using wogonin as a reducing and stabilizing agent, following which the synthesized AgNPs were characterized by various analytical techniques. The synthesized AgNPs exhibited dose- and size-dependent toxicity towards A549 cells. Interestingly, the combination of AgNPs and MS-275 significantly induces apoptosis, which was accompanied by an increased level of reactive oxygen species (ROS); leakage of lactate dehydrogenase (LDH); secretion of TNFα; dysfunction of mitochondria; accumulation autophagosomes; caspase 9/3 activation; up and down regulation of pro-apoptotic genes and anti-apoptotic genes, respectively; and eventually, induced DNA-fragmentation. Our findings suggest that AgNPs and MS-275 induce cell death in A549 lung cells via the mitochondrial-mediated intrinsic apoptotic pathway. Finally, our data show that the combination of AgNPs and MS-275 is a promising new approach for the treatment of lung cancer and our findings contribute to understanding the potential roles of AgNPs and MS-275 in pulmonary disease. However, further study is warranted to potentiate the use of this combination therapy in cancer therapy trials.

## 1. Introduction

Cancer is still a prevalent disease, and lung cancer is one of the most fatal chronic diseases. It has been the second most common cause of cancer-related death in both men and women worldwide, for several decades. Lung cancer is mainly found in elderly people aged 65 or older worldwide. According to a report in 2012, 1,800,000 new lung cancer cases were estimated and diagnosed worldwide and as a result of lung cancer, approximately 1,590,000 people were killed [1,2]. Generally, lung disease is associated with deregulated apoptosis. According to 2012 data, lung cancer killed 24% and 14% of all cancer deaths in males and females, respectively. Recently, nanotechnology-mediated therapy has played an important role in lung cancer by overcoming the current constraints in conventional therapies to prevent severe adverse side-effects on tissue and cells. In addition, conventional chemotherapy treatments include single and non-targeted therapies that can lead to drug resistance [3]. Hence, a combination of histone deacetylases inhibitors (HDACi) and nanoparticles would be a viable alternative approach, by reducing the adverse effects of individual drugs and synergizing their anticancer effects through the combination of different action mechanisms [4,5,6,7,8,9].

It is well known that many tumors are categorized by an imbalance of histone deacetylases (HDACs) and histone acetylases (HAT) activity, which is involved in the remodeling of the chromatin structures and affects the accessibility of the chromatin to transcription factors to start gene transcription. HDACs regulate the expression of genes involved in various cellular functions, including cell proliferation, cell viability, tumor suppression, cell cycles, differentiation, and DNA repair. Hence, HDACi are considered to be a vital class of targets in cancer treatment strategies, and are becoming a new promising class of anticancer drugs [10,11,12]. There are four classes of HDAC inhibitors, among which MS-275 is a promising candidate. It is already in pre-clinical and clinical development, due to its structural diversity, and it can prevent cell cycle progression and induce growth arrest of cancer cells [13,14,15]. MS-275 is selective, has the anticancer properties of HDACi, and has a long serum half-life. MS-275 induced apoptosis of neuroblastoma cell lines after 48 h exposure and significantly reduced growth of adrenal orthotopic xenografts [16]. The combination of IL-2 and MS-275 exhibited synergistic antitumor effects in murine models of renal cells by decreasing the number of T regulatory cells and increasing antitumor cytotoxicity by splenocytes and sensitizes TRAIL-resistant breast cancer cells [17,18]. MS-275 decreased the cell viability and induced differentiation of NB cell lines [19,20]. HDACi exhibited synergistic anticancer effects with conventional chemotherapeutic agents, such as cisplatin and etoposide in neuroblastoma cells [21]. MS-275 and salermide potentiate anti-proliferative effects when combined with EF24 by effectively reducing pancreatic cancer cell (BxPC-3) progression and stopping the cell cycle at the G1 phase [22]. MS-275 can increase senescence in mesenchymal stem cells and decreases the expression of stemness genes [23]. The combination of acetazolamide (AZ) and MS-275 significantly inhibited growth, induced cell cycle arrest and apoptosis, and reduced the migration capacity of SH-SY5Y. Further, the combination reduces tumor growth by a marked reduction in tumorigenicity [23].

Among several metal nanoparticles, silver nanoparticles possess various biological properties and have been used extensively in nanomedicine [24]. Previously, several studies showed that AgNPs possess a cytotoxic activity toward a variety of cancer cell lines and in animal models of cancer following intra-tumoral injection [25,26,27]. AgNPs causes dose- and time-dependent toxicity by generation of oxidative stress; leakage of cytotoxic markers; DNA damage; mitochondrial dysfunctions; and upregulation of pro-apoptotic and down regulation of anti-apoptotic marker genes in several types of cancer cells, including NIH3T3 cells, human lung carcinomas, ovarian, breast, and neuroblastoma cancer cells [28,29,30,31]. AgNPs have been shown to enhance the apoptosis and autophagy efficacy of chemotherapeutic drugs including 5-fluorouracil, doxorubicin, salinomycin, cisplatin, and gemcitabine by creating imbalance of pro-oxidants and anti-oxidants levels in the cellular system in a variety of cancer cells [6,7,32,33,34]. The intracellular reactive oxygen species (ROS) play a major role in the regulation of mitochondrial network dynamics, including mitochondrial distribution, density, activity, and size. The mitochondrion is involved in various functions, such as storing calcium, regulating metabolism, controlling cell death, and cell signaling. Mitochondrial DNA (mtDNA) plays an important role in enzyme production and also provides an energy source for the cell. The malfunctioning of cells is directly related to ineffective mtDNA functioning, which can lead to cellular death.

The cytotoxicity of AgNPs depends on several factors, including size, shape, surface chemistry, surface area, concentration, lateral dimension, surface structure, functional groups, purity and protein corona, type of reducing agent used for synthesis, and finally the type of cancer cells and concentration of materials used for experiments. AgNPs are able to kill mammalian cancer cells within the range between 2–100 μg/mL [27,30,35,36]. To overcome the undesired side effects of using high concentrations of nanoparticles and chemotherapeutic agents by using a minimum level of dose to enhance the cytotoxic performances, we hypothesized that combining AgNPs with MS-275, a potent selective HDACi, would be more effective than either single agent alone against lung carcinoma cells. Therefore, this study was designed to evaluate the combination effect of AgNPs and MS-275 in human alveolar basal epithelial cells.

## 2. Results and Discussion

### 2.1. Synthesis and Characterization of Silver Nanoparticles Using Wogonin

**Figure 1:**
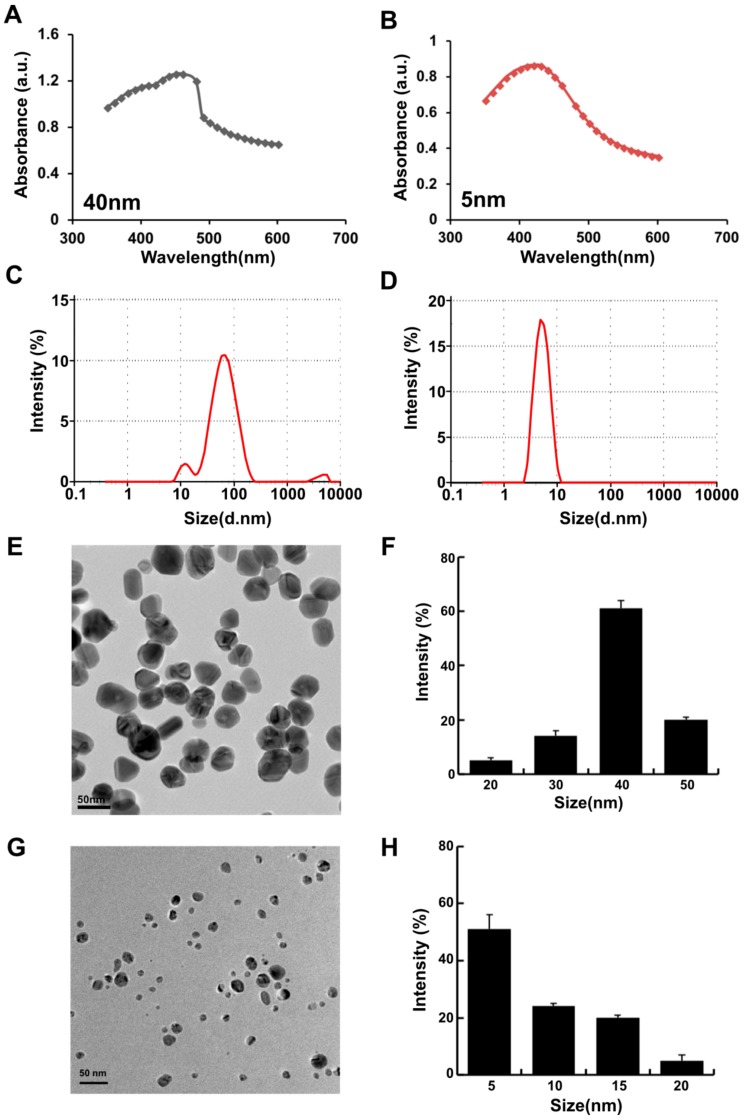
Synthesis and characterization of AgNPs using wogonin. (**A**,**B**) UV-visible (vis) spectrum of 40 nm and 5 nm AgNPs. (**C**,**D**) Size distribution analysis of 40 nm and 5 nm AgNPs. (**E**) Transmission electron microscopy (TEM) images of 40 nm size of AgNPs. (**F**) Histogram showing size distributions based on TEM images of AgNPs ranging from 20 to 50 nm with an average size of 40 nm. (**G**) TEM images of 5 nm size of AgNPs. (**H**) Histogram showing size distributions based on TEM images of AgNPs ranging from 5 to 20 nm with an average size of 5 nm.

Recently, synthesis of AgNPs was carried out by green method for controlling the size and shape, stability, and solubility. The green synthesis approach was established using a variety of reducing agents, including a cellular extract of microbes; purified enzymes; plant extracts; and purified phenolic compounds, such as quercetin [7,37,38,39]. In this experiment, we explored the possibility of using the unexplored O-methylated flavonoid called wogonin, which is found in *Scutellaria baicalensis* [40]. In our experiment, we have used purified wogonin for the synthesis of AgNPs to eliminate unnecessary contaminants in the cellular assays. The wogonin-mediated synthesis of AgNPs was performed by using two different concentrations of wogonin (1 and 5 mg/mL) with 1 mM AgNO_3_ at 40 and 60 °C at pH 8.0 and 10.0, respectively. The rate of synthesis and color formation was higher at 60 °C compared with that at 40 °C, which is due to the increased temperature allowing particle growth at a higher rate; moreover, it is favorable for the synthesis of smaller-sized particles [26]. The color change is attributed to the changes in the size and morphology of the AgNPs with time. The excitation of surface plasmonresonance caused by the reduction reaction was analyzed using UV/Vis (visible) spectroscopy (Biochrom, Cambridge, UK); the spectra showed peaks at wavelengths of 420 and 400 nm (Figure 1A). Furthermore, the size distribution was confirmed by dynamic light scattering (DLS) analysis (Zetasizer Nano ZS90, Malvern Instruments Limited, Malvern, WR, UK). The synthesis of the smaller size of the particle depends on various factors such as temperature, pH, concentration of reducing agent, and concentration of AgNO_3_. Smaller size particles can be achieved at high temperature and increasing concentration of AgNO_3_. As a result, the combination of 1 mg/mL wogonin with 1 mM AgNO_3_ at 40 °C produced particles with an average size of 40 nm, and 5 mg/mL wogonin with 1 mM AgNO_3_ at 60 °C at pH 10.0 produced particles with an average size of 5 nm (Figure 1B). Further, we confirmed the size and shape of the particles by transmission electron microscopy (TEM). DLS analysis revealed that two different concentrations of wogonin at 40 and 60 °C produced particles with an average size of 40 and 5 nm, respectively (Figure 1C,D), which is in agreement with the TEM size and morphology of TEM micrographic images shows at 40 nm (Figure 1E,F) and 5 nm (Figure 1G,H). The synthesized nanoparticles seem to be polydispersity in nature. The produced nanoparticles show polydispersity in nature. A nanoparticle system with PDI value < 0.1 is considered as highly monodisperse, while PDI value > 0.4 and value in range of 0.1–0.4 are indications that the system has highly polydisperse and moderately disperse distribution, respectively [41]. The prepared AgNPs shows an average size of 40 and 5 nm with PDI value of 0.112 and 0.119, respectively, which indicates that the prepared AgNPs are monodisperse in nature.

### 2.2. Size-Dependent Toxic Effect of AgNPs on Cell Viability of A549 Cells

**Figure 2:**
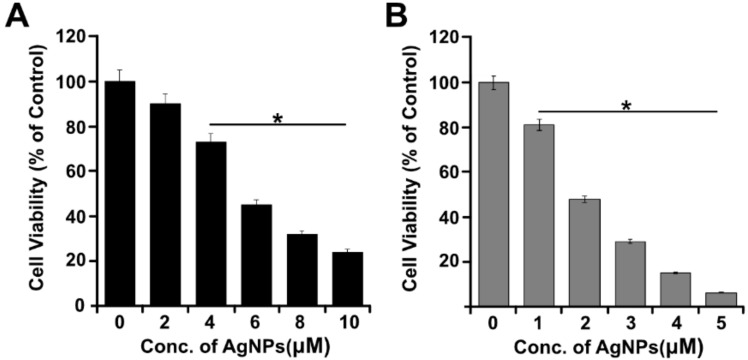
Dose-dependent effect of 40 and 5 nm AgNPs in A549 cells. (**A**) A549 cells were incubated with various concentrations of 40 nm AgNPs (2–10 μM) for 24 h, and cell viability was measured using CCK-8. (**B**) A549 cells were incubated with various concentrations of 5 nm AgNPs (1–5 μM) for 24 h, and cell viability was measured using CCK-8. The results are expressed as the mean ± standard deviation of three separate experiments. Statistically significant differences between the treated and control group are indicated by (* *p* < 0.05).

A549 cells were exposed to two different sizes of AgNPs, 40 nm particles with concentrations of 2–10 μM and 5 nm particles with concentrations 1–5 μM, for 24 h. After 24 h, significant signs of toxicity were observed for both sizes of AgNPs up to the highest dose tested. Significant cell toxicity (*p* ≤ 0.05) was observed for the 40 nm particles above 4 μM, whereas significant toxicity (*p* ≤ 0.05) was observed for the 5 nm AgNPs even at 1 µΜ concentration. The increasing concentration of AgNPs had a pronounced effect on cell viability for both the smaller and larger particles (Figure 2A). The results show that both sizes of the AgNPs lead to dose-dependent inhibition on cell viability. To measure the effectiveness of the two different sizes of AgNPs, 50% inhibitory concentration (IC_50_) was calculated. The IC_50_ of the 40 nm was found to be 6 (Figure 2A) and IC _50_ of the 5 nm was found to be 2 μM (Figure 2B). As a result, the smaller-size AgNPs with an average size of 5 nm caused significant toxicity compared with the 40 nm particles, which could be due to the cellular uptake of AgNPs. For example, Jiang et al. [42] reported that the size dependent cellular uptake of AgNPs of 20 nm was greater than that of AgNPs of 100 nm in human glioma U251 cells. Among the three different sizes of AgNPs tested, that is, 20, 80, and 113 nm, the 20 nm particles caused significant cytotoxicity, inflammation, genotoxicity, and developmental toxicity [43]. Our findings also suggest that among two different size of the particles, smaller size of particle, that is, 5 nm, exhibited severe toxicity with low IC_50_ value of 2 μM rather than 6 μM of 40 nm particles. Therefore, we selected 5 nm-sized AgNPs for further experiments. The toxicity was observed with average particle size. The toxicity also depends on particles retention and excretion from the tissues. For instance, 5 nm particles can be removed by kidney and larger particles excreted through urine [44,45]. Previous studies also found that silica NPs larger than 100 nm can be rapidly excreted from urine [44,45]. Further, He et al directly observed fluorescent silica NPs with diameters of 45 nm in urine by TEM. 

### 2.3. Effect of Acetamide and MS-275 on Cell Viability of A549 Cells

**Figure 3:**
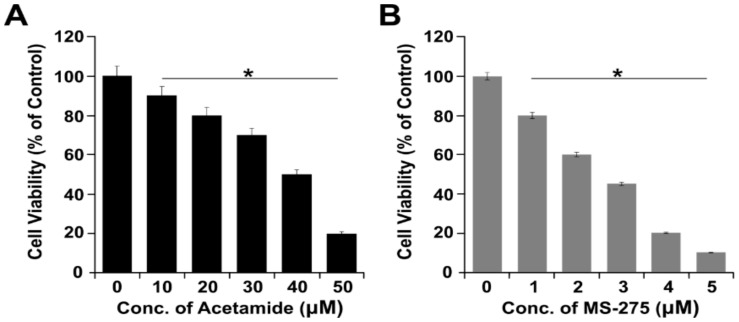
Dose-dependent effect of acetamide and MS-275 in A549 cells. (**A**) A549 cells were incubated with various concentrations of acetamide (10–50 μM) for 24 h, and cell viability was measured using CCK-8. (**B**) A549 cells were incubated with various concentrations of MS-275 (1–5 μM) for 24 h, and cell viability was measured using CCK-8. The results are expressed as the mean ± standard deviation of three separate experiments. Statistically significant differences between the treated and control group are indicated by (* *p* < 0.05).

To determine the efficacy of acetamide and MS-275, we performed a cell viability assay using CCK-8. A549 cells were exposed to different concentrations of acetamide (10–50 μM) and MS-275 (1–5 μM) for 24 h, and cell survival rates were calculated. Although both the tested candidates inhibited cell growth of A549, MS-275 showed more effective killing of A549 cells. To measure the effectiveness of the two different HDACi, IC_50_ was calculated. The IC_50_ of acetamide and MS-275 was 40 and 3 μM, respectively. The IC_50_ values of acetamide and MS-275 were distinct and comparable. The IC_50_ value of acetamide was higher compared with that of MS-275, as reported earlier, and it requires a higher drug concentration to have a significant effect [12,46]. The significant difference in IC_50_ values for acetamide and MS-275 indicates that MS-275 is more potent than acetamide (Figure 3). The antineoplastic effects of three different HDAC inhibitors on growth and apoptosis of the gastrointestinal NET cell lines CM and BON were evaluated using trichostatin A (TSA), sodium butyrate (NaB), and MS-275. The growth inhibition was observed to be dose-dependent, with the inhibition of proliferation of both cell lines with IC_50_ values in the millimolar range for NaB, micromolar range for MS-275, and nanomolar range for TSA. Therefore, we selected the effective HDACi, MS-275, for further combinatorial experiments with AgNPs. 

### 2.4. Combination Effect of AgNPs and MS-275 on Cell Survival

**Figure 4:**
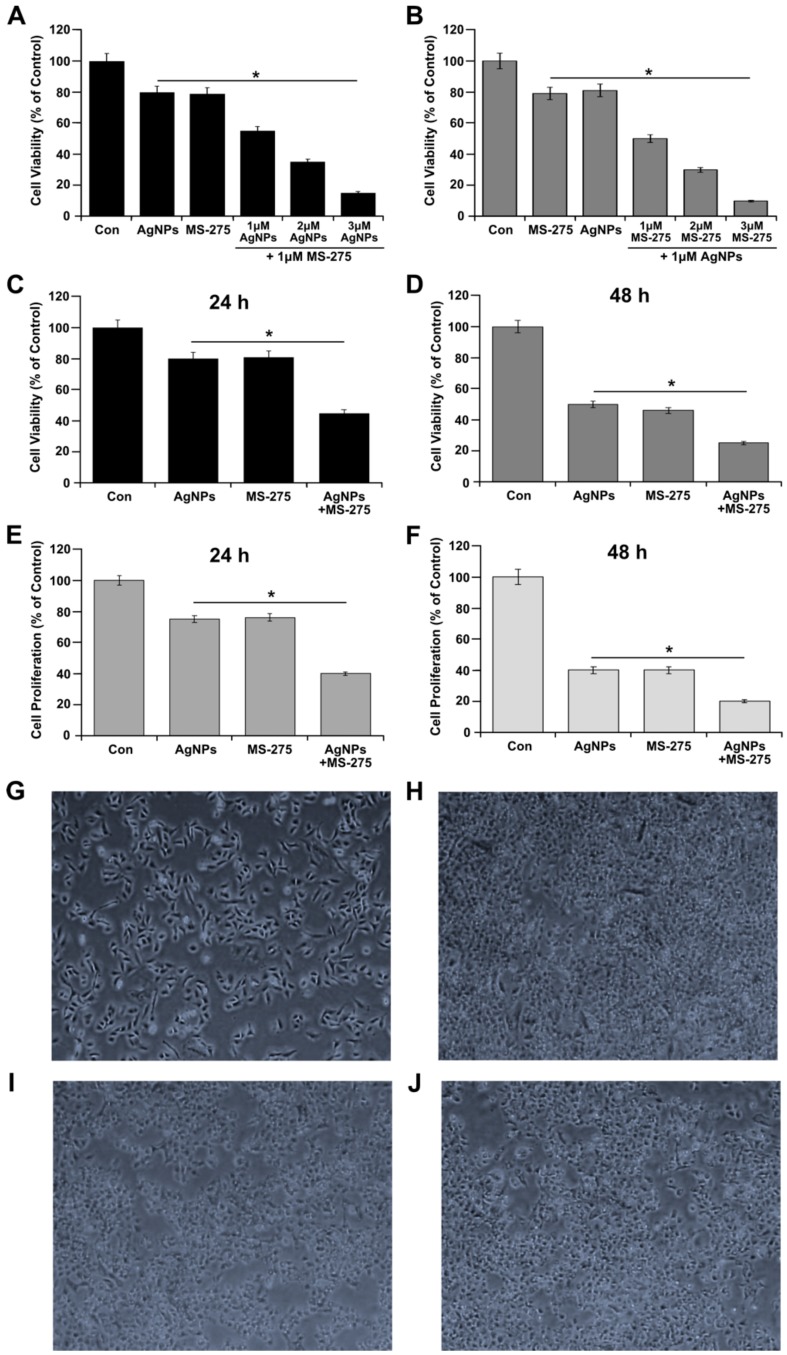
Effect of combination of AgNPs and MS-275 on cell viability in A549 cells. (**A**) A549 cells were incubated with a combination of different concentrations of AgNPs (1–3 μM) and a fixed concentration of MS-275 (1 μM) for 24 h and cell viability was measured using CCK-8. (**B**) A549 cells were incubated with a combination of different concentrations of MS-275 (1–3 μM) and a fixed concentration of AgNPs (1 μM) for 24 h. (**C**,**D**) Cell viability and (**E**,**F**) cell proliferation was determined in A549 cells were incubated with AgNPs (1 μM), MS-275 (1 μM), or a combination of AgNPs (1 μM) and MS-275 (1 μM). (**G**–**J**) Cell morphology was assessed under a light microscope. (G-control; H-AgNPs; I-MS-275; J-AgNPs; and MS-275). The results are expressed as the mean ± standard deviation of three separate experiments. Differences between the treated and control groups were measured using Student’s *t*-test. Statistically significant differences between the treated and control group are indicated by (* *p* < 0.05).

First, to determine the optimum concentration for a better combination effect, clinically acceptable concentrations of AgNPs and MS-275 were selected. Further, to avoid undesired toxic effects, A549 cells were treated with different combinations of AgNPs and MS-275. We examined the effect of the simultaneous addition of AgNPs (1, 2, and 3 μM) with a fixed concentration of MS-275 (1 μM) in A549 cells. Conversely, to gain the consistency in the combination experiments, we simultaneously added MS-275 (1, 2, and 3 μM) with a fixed concentration of AgNPs (1 μM) in A549 cells. The results show that increasing concentrations of either AgNPs or MS-275 reduce the cell viability significantly; however, at a low concentration of the combination of AgNPs and MS-275, significantly lower cell viability than higher concentrations of individual AgNPs or MS-275 was observed, showing a remarkable effect. This indicates that a lower concentration of AgNPs and MS-275 is sufficient to produce synergistic action rather than using higher concentrations to induce cell death in A549 cells (Figure 4A). The low concentration of AgNPs and MS-275 is a physiologically acceptable concentration. Figure 4A shows that both AgNPs and MS-275 had a more significant concentration-dependent inhibitory effect on A549 cells, whereas the AgNPs or MS-275 were less effective when used alone. The combination effect was significantly enhanced (Figure 4B). Therefore, further combination experiments were carried out using 1 μM of AgNPs and MS-275. Our findings are consistent with a previous report that demonstrated that AgNPs could inhibit the cell survival in a variety of cancer cells, including normal (L132) and human lung cancer (A549), ovarian (A2780), human breast cancer cells (MCF-7 and MDA-MB 231), and male- and female-derived somatic and germ cells [29,47].

To further evaluate the combination effect of AgNPs and MS-275, we used CCK-8 and BrdU, representing metabolic mitochondrial effects and proliferation of cells, respectively [46]. The cells were treated with AgNPs (1 μM), MS-275 (1 μM), and a combination of AgNPs (1 μM) and MS-275 (1 μM), and cell viability and proliferation were evaluated after 24 and 48 h. The results showed that the combination of AgNPs and MS-275 exhibited a significant time-dependent effect in cell viability of cells compared with AgNPs or MS-275 alone (Figure 4C,D). To corroborate the results obtained from the cell viability assay, we performed cell proliferation assay to the combination effect of AgNPs and MS-275 on cell proliferation for 24 h treated cells. The A549 cells were treated with AgNPs (1 μM), MS-275 (1 μM), and a combination of AgNPs (1 μM) and MS-275 (1 μM). As shown in Figure 4E,F, AgNPs and MS-275 inhibited 50% cell proliferation even at a low dose of 1 μM (*p* < 0.05). The cell proliferation was significantly reduced to 50% at 1 μM by AgNPs or MS-275. It was concluded that both AgNPs and MS-275 inhibited cell proliferation dramatically compared with a single agent alone, the greatest potential toxicity was observed with combination. Finally, we selected AgNPs (1 μM) and MS-275 (1 μM), and 24 h treatment time for the rest of the study. 

After confirmation of the effectiveness of the combination of AgNPs (1 μM) and MS-275 (1 μM) on cell viability and cell proliferation, to further prove the effectiveness of the dose combination, we assessed the morphological features by monitoring the cell morphology of A549 cells, which is a preliminary checkpoint for apoptosis. A549 cells were treated with AgNPs (1 μM), MS-275 (1 μM), and a combination of AgNPs (1 μM) and MS-275 (1 μM) for 24 h, and then images were recorded. As shown in Figure 4G, control cells had a normal cell morphology with a distinct cell membrane, and most of the normal cells were scattered in the whole field of the microscope. The cells treated with AgNPs (1 μM) and MS-275 (1 μM) aggregated and were detached from the cell surface (Figure 4H–J). On the other hand, cells challenged by the combination of AgNPs (1 μM) and MS-275 (1 μM) exhibited cell shrinkage, condensed cytoplasm, an increased percentage of the nucleus, as well as formation of elongated cellular protrusions. Morphological analysis showed that more cell death was induced after the combination treatment with AgNPs (1 μM) and MS-275 (1 μM) than those of either individually. These results suggest that AgNPs or MS-275 could increase the cell death by increasing oxidative stress. Similarly, human cervical cancer cells (HeLa cells) were treated with 1.5 μM TSA or 2 μM vorinostat to show a typical morphology characterized by elongated protrusion, and further TSA and vorinostat decrease the cell viability [48]. Collectively, cell viability, cell proliferation, and cell morphology data indicate that AgNPs potentiate the cytotoxic effect of MS-275 in A549 cells. 

### 2.5. AgNPs and MS-275 Enhances Cytotoxicity 

**Figure 5:**
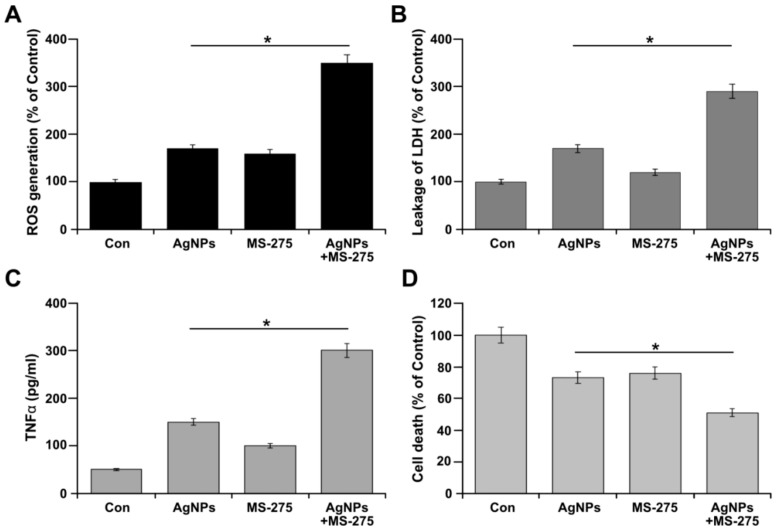
AgNPs and MS-275 induce cytotoxicity. A549 cells were incubated with AgNPs (1 μM), MS-275 (1 μM), or a combination of AgNPs (1 μM) and MS-275 (1 μM) for 24 h. (**A**) The levels of reactive oxygen species (ROS) were assessed by measuring the relative fluorescence of 2′,7′-dichlorofluorescein using a spectrofluorometer. (**B**) The activity of lactate dehydrogenase (LDH) was measured at 490 nm using the LDH cytotoxicity kit. (**C**) Measurement of induction of TNFα. (**D**) The level of dead-cell protease was determined by CytoTox-Glo cytotoxicity assay. The results are expressed as the mean ± standard deviation of three independent experiments. Statistically significant differences between the treated and control group are indicated by (* *p* < 0.05).

AgNPs and HDACi are known to induce cytotoxicity through oxidative stress; leakage of the cell death marker lactate dehydrogenase (LDH), secretion of pro-inflammatory markers such as TNFα, and—as a consequence of cytotoxicity—cell viability loss was observed and eventually leads to cell death. Hence, we determined the effect of AgNPs (1 μM), MS-275 (1 μM), and a combination of AgNPs (1 μM) and MS-275 (1 μM) on oxidative stress by measuring the ROS level. As we expected, the treated cells exhibited significantly higher levels of ROS than the untreated cells (Figure 5A). Interestingly, the combined treatment resulted in a four-fold increase in ROS compared with the untreated group, whereas a two-fold increase was observed for treatment with AgNPs or MS-275 alone. Engineered nanomaterials are capable of inducing toxicity by generation of ROS through interaction with cells, and this could be a feasible and reliable mechanism of toxicity [49]. Several studies substantiated that the toxicity of AgNPs in a variety of cancer cells exposed to AgNPs leads to increased intracellular ROS levels [27,30]. Thus, oxidative damage caused by increased ROS is thought to contribute to AgNPs-mediated cell death in A549 cells. Similarly, one study showed that HDAC increases the levels of ROS and activating the mitochondrial apoptotic pathway promotion of DNA damage [50].

Several studies demonstrate that AgNPs elevate intracellular level of ROS and alter redox-homeostasis of cancer cells. It is widely accepted that the anticancer effect of AgNPs is due to induction of oxidative stress and ROS-mediated apoptosis in cancer. However, a study has reported induction apoptosis by an ROS-independent mechanism. For example, Jacobson and Raff [51] have shown that apoptosis has occurred when the cells are cultivated in hypoxic conditions without presence of ROS. Shimizu et al. [52] have failed to detect ROS during hypoxia-induced apoptosis. This evidence suggests that ROS are not obligator effectors in the apoptosis. The mechanism of apoptosis by ROS independently is induced by a decrease of mitochondrial functions and Bcl-2/Bax ratio, translocation of cytochrome *c* from the mitochondria into the cytosol, and activation of caspases 3 and 9 [53]. Therefore, we further confirmed the apoptotic response of AgNPs by DNA fragmentation and caspase activity in AgNPs or MS-275 or a combination of AgNPs and MS-275. 

The typical cytotoxic marker LDH, which is a cytoplasmic enzyme released into the media, indicates cytotoxicity. Incubating cells with AgNPs and MS-275 for 24 h treatment resulted in significant increases of LDH release compared with single treatment. This indicates that the AgNPs and MS-275 directly affect the cell membrane integrity. LDH release showed nearly a three-fold increase compared with the control levels (Figure 5B). This was most likely because of the loss of cell membrane integrity of the cells in late apoptotic stages. Our results are consistent with a previous study, in which it was found that increasing the concentration of various HDACi increases LDH release in CM and BON cells, depending on the concentration of HDACi and type of cells [54]. HDACi, vorinostat, and BML281 induce leakage of LDH on GM-CSF- and M-CSF-derived primary human macrophages in a concentration-dependent manner [55]. Similarly, the combination of palladium nanoparticles with trichostatin A and AgNPs with gemcitabine increased LDH in human breast cancer cells and ovarian cancer cells [7,24]. 

Measuring the lactate dehydrogenase (LDH) is a useful method for detection of necrosis. LDH release assay as a consequence of apoptotic cell death. To distinguish necrosis versus apoptosis, we performed caspase activity and DNA fragmentation in AgNPs treated cells. The results from our study indicate that AgNPs or MS-275 or a combination of AgNPs and MS-275 induces cytotoxicity on human lung cancer cells, although we could not distinguish between necrosis and apoptosis. In order to clarify whether the AgNPs/MS-275-induced cytotoxic effect was due to a necrotic and/or apoptotic mechanism, several parameters associated with both mechanisms were studied. First, we measured AgNPs/MS-275 induced LDH release, which also suggests a necrotic process. The LDH release was higher when the exposure to AgNPs/MS-275 was performed in the absence of AgNPs/MS-275. Under these conditions, the exposure to 1 µM of AgNPs or MS-275 for 24 h was enough to release LDH and induce necrosis dependent cell death. The AgNPs-induced necrosis accompanied by LDH release has been found in several cell types’ epidermoid larynx carcinoma cells and human lung cells [56,57]. Indeed, in our experiment, LDH leakage was detected when human lung cells were exposed to AgNPs, indicating that the toxic effects observed were due to AgNPs. Collectively, our findings suggest that that necrosis can also function as an alternative programmed mode of cell death, triggered by the same death signals that induce apoptosis.

TNFα is considered to be an important secretion factor to stimulate several signaling pathways leading to inflammation, apoptosis, and tissue degradation [58,59]. In this study, the cells treated with AgNPs and MS-275 showed a two-fold increased level of TNFα secretion compared with the single agents, and a dramatic increase around six-fold was observed compared with the untreated control group (Figure 5C). Alveolar macrophages treated with 15 nm AgNPs showed significant inflammatory response, observed by the release of TNFα, MIP-2, and IL-1β [58,60]. HDACs, vorinostat, and BML281 modulate cytokine are overexpressed by suppressing the release of inflammatory mediators, such as IL-12, p40, and IL-6, at low concentrations (<3 μM), but they increase the production of other cytokines including TNF and IL-1βat higher concentrations (>3 μΜ), such as IL12, p40, and IL6 in human macrophages, which indicate that pro-inflammatory responses are concentration-dependent [55]. Our findings clearly suggest that the combination of AgNPs and MS-275 significantly potentiates the production of TNFα in A549 cells compared with single treatment.

TNFα plays critical role in pro-inflammatory responses. Apoptosis may be triggered by extrinsic stimuli through various cell surface death receptors [61]. This is the first study we interested to correlate the relationship between ROS and TNFα to increase the cytotoxicity in human lung cancer. Previous studies reported that uncontrolled generation of ROS triggers a cascade of pro-inflammatory cytokines and mediators via activation of redox sensitive MAPK and NF-κB signaling pathways that control transcription of inflammatory genes such as IL-1β, IL-8, and TNF-α [62], and oxidative stress also plays a key role in NP-induced airway hypersensitivity and respiratory inflammation [63]. SiO_2_ and TiO_2_ nanoparticles induce an elevated inflammatory response through the underlying mechanism of ROS generation [64,65,66]. Therefore, study also indicates AgNPs not only acting anti-inflammatory, but also pro-inflammatory, which depends on concentration of AgNPs and type of cells, and further, the cross between ROS and TNF-alpha could induce cytotoxicity in cancer cells particularly at given dose. 

Finally, cell death protease activity, which is associated with cell death or viability parameters, was assessed to determine the ratio of cell death/viability. These assays generate large dynamic ranges with excellent linearity, providing unprecedented sensitivity. The cell death ratio of the combined AgNPs and MS-275 treated group significantly increased (49%) compared with that of single treatment (27%) (Figure 5D). These results suggest that AgNPs potentiate the growth inhibitory effects stimulated by MS-275 on A549 cells. Similarly, the combination of AgNPs with gemcitabine increase the cell death ratio compared with single treatment with either AgNPs or gemcitabine in human ovarian cancer cells [7]. Mokhtari et al. [46] evaluated the antitumor potential of the HDAC inhibitor (HDACi), pyridylmethyl-*N*-{4-[(2-aminophenyl)-carbamoyl]-benzyl}-carbamate (MS-275), in combination with a pan CA inhibitor, acetazolamide (AZ), on NB SH-SY5Y, SK-N-SH, and SK-N-BE(2) cells. They found that the combination of AZ and MS-275 significantly inhibited cell growth, induced cell cycle arrest and apoptosis, and reduced the migration capacity of the NB cell line SH-SY5Y. Collectively, all these cytotoxic assays revealed that AgNPs potentiate the toxicity of MS-275 in A549 cells. The combination index (CI) was used to evaluate the combination effect based on IC_50_ values obtained from AgNPs or MS-275 alone and a combination of both. Our results indicated that all combinations of AgNPs with MS-275 gave rise to CI values significantly below 1 at fa (GI) = 0.5, indicating synergistic effect.

### 2.6. Effect of AgNPs and MS-275 on Oxidative and Anti-Oxidative Stress Markers

**Figure 6:**
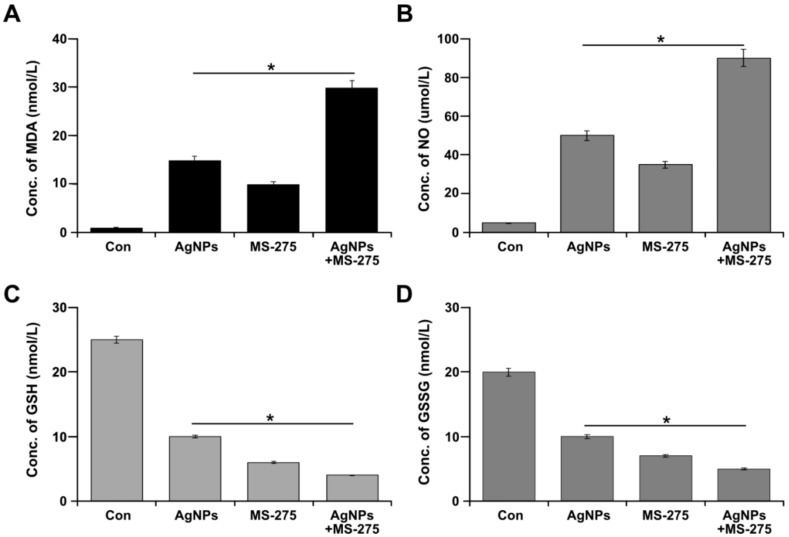
Combination effect of AgNPs and MS-275 on pro and anti-oxidant markers in A 549 cells. A549 cells were incubated with AgNPs (1 μM), MS-275 (1 μM), or combinations of AgNPs (1 μM) and MS-275 (1 μM) for 24 h. (**A**) The concentration of MDA was measured and expressed as nanomoles per milliliter. (**B**) NO production was quantified spectrophotometrically using the Griess reagent and expressed as micromoles per milliliter. (**C**) GSH concentration is expressed as nanomoles per milliliter. (**D**) GSSG concentration is expressed as nanomoles per milliliter. Results are expressed as mean ± standard deviation of three independent experiments. There was a significant difference in treated cells compared with untreated cells with Student’s *t*-test (* *p* < 0.05).

Oxidative stress is the result of the imbalance between pro- and antioxidant levels in the cellular system. The precise functionality and cellular vital functions depends on the redox equilibrium of cells [67]. Owing to reactive oxygen and nitrogen species (ROS/RNS), several cellular biomolecules, including lipids, sugars, proteins, and polynucleotides, cause damage. Therefore, cells try to prevent cell damage by increasing non-enzymatic molecules such as glutathione, as well as enzymatic scavengers of ROS, with superoxide dismutase (SOD), catalase (CAT), and glutathione peroxidase (GPX) [67,68]. Therefore, we examined the level of pro-oxidants markers, such as MDA and NO, and anti-oxidant markers, such as GSH and GSSG, in cells treated with AgNPs (1 μM), MS-275 (1 μM), and a combination of AgNPs (1 μM) and MS-275 (1 μM). The results revealed that the level of MDA and NO significantly increased in the treated cells (Figure 6A,B). Interestingly, the combined treatment shows a remarkable elevation compared with that using untreated or single treatment cells. Conversely, the level of antioxidants such as GSH and GSSG was significantly lower in the combined AgNPs (1 μM) and MS-275 (1 μM) treated cells than either AgNPs or MS-275 alone treated cells or the untreated group (Figure 6C,D). Similarly, the combination of AgNPs and salinomycin or AgNPs and gemcitabine increased various oxidative stress markers and decreased antioxidative stress markers in human ovarian cancer cells [6,7]. AgNPs seem to have a strong affinity for thiol groups, such as GSH, and they can easily deplete the GSH levels in cells; the decreased level of GSH has been shown to increase the cytotoxicity of AgNPs [35,69]. For instance, the reduction of cellular GSH levels increases the sensitivity of neurons to toxic insults and induces changes in mitochondrial function [70]. Govender et al. [71] observed that, due to strong affinity of AgNPs for thiol (-SH) groups, the levels of cysteine-rich GSH were decreased, while lipid peroxidation was significantly elevated by AgNPs. This oxidant/antioxidant imbalance has a critical role in the apoptotic mechanism by AgNPs-mediated cytotoxicity [72,73,74]. Thus, the toxicity merely depends on the level of pro-oxidants and antioxidants in cellular system. 

### 2.7. Effects of AgNPs and MS-275 on Cell Structure 

**Figure 7:**
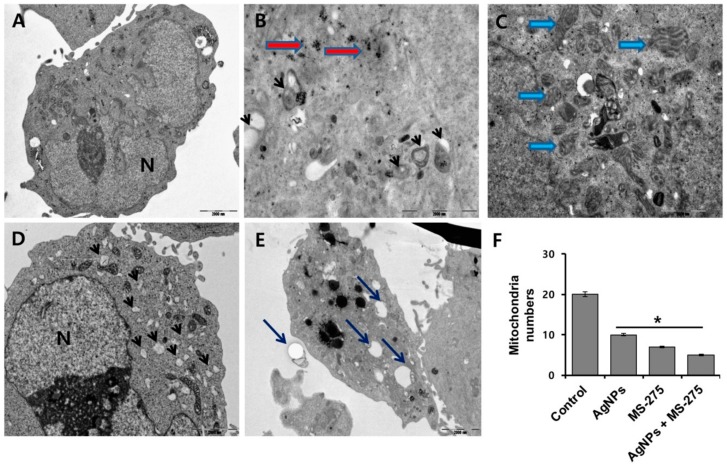
AgNPs and MS-275 induced accumulation of autophagosomes. A549 cells were incubated with AgNPs (1 μM), MS-275 (1 μM), or a combination of AgNPs (1 μM) and MS-275 (1 μM) for 24 and hand-processed for TEM. (**A**) The control cells showed clear nuclear morphology without any defects in organelles. (**B**) AgNPs treated cells depicting AgNPs enter into the cells and cause accumulation of autophagosomes (thick short arrow). (**C**) MS-275-treated cells showed accumulation of autophagosomes, swollen mitochondria, and many multi-vesicular and membrane-rich autophagosomes. (**D**) AgNPs and MS-275-treated cells showed accumulation of a significant number of autophagosomes. (**E**) Mitochondria copy number was determined by real-time PCR. (**F**) Results are expressed as mean ± standard deviation of three independent experiments. There was a significant difference in treated cells compared to untreated cells with Student’s *t*-test (* *p* < 0.05).

Autophagy is a dynamic self-digestion process, which comprises initiation, elongation, fusion, and degradation processes. Autophagy comprises the processes of autophagosome synthesis and lysosomal degradation; accumulation autophagosomes can be associated with loss of cell viability [75]. Various stress conditions concurrently induce both compromised autolysosomal activity and increased autophagosome synthesis [76]. The increased accumulation of autophagosomes occurs mainly through the inhibition of autophagosomes–lysosomes fusion and dysfunction of lysosomes. To investigate the effect of AgNPs and MS-275-induced autophagosome accumulation and the size of mitochondria, A549 cells were treated with AgNPs (1 μM), MS-275 (1 μM), and a combination of AgNPs (1 μM) and MS-275 (1 μM) for 24 h. The control cells showed a clear nucleus morphology and distinct cell membrane (Figure 7A). The cells treated with AgNPs clearly indicated the entry of AgNPs into the A549 cells (red color arrow) occurred, and AgNPs were clearly observed inside the cells (Figure 7B). As a result, the entry of AgNPs into the cells caused accumulation of double membrane vesicles called autophagosomes (thick black bold arrow) (Figure 7B). Interestingly, MS-275 caused accumulation of autophagosomes and also increased the size of the mitochondria, and eventually the numbers of mitochondria were reduced per cell (Figure 7C). This is consistent with a previous study in which it was found that AgNPs-treated human neuroblastoma cells exhibited an increased mitochondria size and decreased number of mitochondria [77]. Interestingly, MS-275 induced accumulation of a significant number of autophagolysosomes (Figure 7D). The combination of AgNPs and MS-275 increased the autophagolysosomes (Figure 7E). These findings suggest that the combination of AgNPs and MS-275 dramatically increases the accumulation of autophagosomes and swollen mitochondria and significantly decreases the number of mitochondria (Figure 7F). Our findings suggest that production/accumulation of autophagosomes subsequently unfused to lysosomes induces cellular toxicity, which are agreement with a previous report stating that the combination of salinomycin and AgNPs increased the accumulation of autophagosomes and autolysosomes compromises cell viability in human ovarian cancer cells. 

Further, we investigated the impact of AgNPs and MS-275 on ROS-induced mitochondrial dysfunction by measuring the mitochondria copy number, which could influence the physiology of cells [78]. To determine the mtDNA copy number, the cells were treated with AgNPs (1 μM), MS-275 (1 μM), and a combination of AgNPs (1 μM) and MS-275 (1 μM) for 24 h, and then the mtDNA copy numbers were assessed. Remarkably, we found that AgNPs and MS-275 treated cells had significantly lower mtDNA copy numbers compared with that of AgNPs or MS-275-treated and untreated cells (Figure 7E). Our findings suggest that both AgNPs and MS-275 may be involved in mitochondrial biogenesis by regulating genes involved in mitochondrial biogenesis. In contrast, Sitarz et al. [79] reported that valproic acid triggers increased mitochondrial biogenesis in POLG-deficient fibroblasts by altering the expression of several mitochondrial genes. With X-ray irradiation in the mitochondria in cells with and without cytoplasmic gold nanoparticles, mitochondrial DNA was subject to significant damage, rather than cytoplasmic DNA [80]. Disruption of the mtDNA copy number could influence the mitochondrial function and could in turn have a significant impact on the general functions of the cells.

### 2.8. AgNPs and MS-275 Cause Mitochondrial Sysfunction in A549 Cells

**Figure 8:**
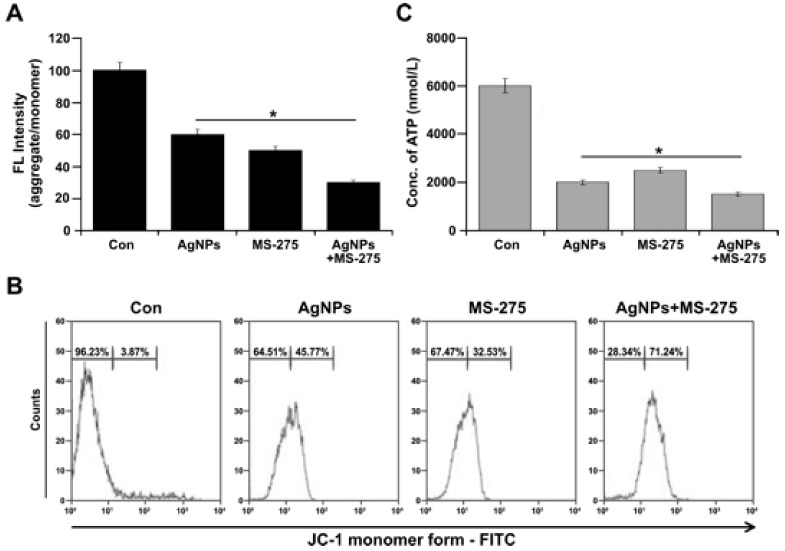
AgNPs and MS-275 cause mitochondrial dysfunction. (**A**) A549 cells were incubated with AgNPs (1 μM), MS-275 (1 μM), or a combination of AgNPs (1 μM) and MS-275 (1 μM) for 24 h, and the mitochondrial membrane potential was determined using the cationic fluorescent indicator JC-1. The loss of membrane potential was represented as aggregate/monomer ratio. (**B**) JC-1 monomer formation was measured using flow cytometry. (**C**) The concentration of ATP was determined by nanomoles per milliliter. The results represent the means of three separate experiments, and error bars represent the standard error of the mean. Treated groups showed statistically significant differences from the control group with Student’s *t*-test (* *p* < 0.05).

The oxidative stress-mediated mitochondrial dysfunction by AgNPs and MS-275 led to the question of whether AgNPs and MS-275 indeed influence mitochondrial function, as suggested by the previous experiments, which revealed swollen mitochondria and decreased number of mtDNA. Mitochondria are known to be involved in various cellular functions, such as cellular metabolism, and disruption or dis-regulation of their functions can lead to significant alterations in cellular energy transport, ATP production, mitochondrial morphology, replication, and levels of oxidative stress within the cell. To determine the effect of AgNPs and MS-275 on mitochondrial membrane potential, A549 cells were treated with AgNPs (1 μM), MS-275 (1 μM), and a combination of AgNPs (1 μM) and MS-275 (1 μM) for 24 h, and then mitochondrial membrane potential (MMP) was measured by using a cationic fluorescent indicator, JC-1, as a marker for intact MMP. Subsequently, JC-1 signals were analyzed. The potential difference across the mitochondrial membrane was significantly reduced in cells treated with AgNPs (1 μM), MS-275 (1 μM), and a combination of AgNPs (1 μM) and MS-275 (1 μM), as indicated by decreased level of aggregate/monomer ratio (Figure 8A). The mitochondrial membrane potential was reduced faster and to a greater extent in cells treated with AgNPs and MS-275 than for the single treatment or untreated group. A recent study suggested that the induction of ROS and alterations in mitochondrial membrane permeability were possible mechanisms by which AgNPs exerted its toxic effects in A549 cells [81]. Recently, Bao et al. [82] demonstrated that quisinostat increased reactive oxygen species (ROS) production and destroyed mitochondrial membrane potential (ΔΨm), inducing mitochondria-mediated cell apoptosis in non-small-cell lung cancer (NSCLC) cells. Further, our data was confirmed by flow cytometry analysis. The findings suggest that consistently, treatment with AgNPs resulted in a high JC-1 monomer/aggregate ratio, indicating the structure of mitochondrial membrane was damaged (Figure 8B). These results suggest that exposure of A549 cells induced mitochondria-mediated apoptosis. Collectively, this finding suggests that the combination of AgNPs and MS-275 potentially causes mitochondrial dysfunction by reducing mitochondrial membrane potential and induces mitochondrial-mediated apoptosis. 

Most anticancer agents induce DNA damage, which initiates the cell death pathways of necrosis and apoptosis, but there is no clear idea about the impact of AgNPs and MS-275 on ATP production, which is important for cellular function. Limiting ATP production will injure the cell, particularly by inhibiting DNA repair following DNA damage induced by anticancer agents [83]; further, the depletion of cellular ATP is another marker of early apoptosis. Therefore, it is necessary to determine the role of AgNPs and MS-275 on ATP levels. The findings from this study suggest that the level of ATP is dramatically reduced in the cells exposed to AgNPs and MS-275 compared with the single agent or untreated cells (Figure 8C). Generally, DNA-damaging agents cause cell death by either necrosis or apoptosis. For instance, severe ATP depletion causes necrosis, whereas ATP is necessary for the initiation and progression of apoptosis induced by a DNA-damaging agent and the activation of caspase-9,8 and for chromatin condensation in apoptosis. At this juncture, ATP needs Vs—ATP depletion mediated cell death event could take place simultaneously in AgNPs and MS-275 treated cells. The loss mitochondrial membrane potential leads to declines in mitochondrial ATP production as the result of a decrease in the proton gradient that drives H^+^ through ATP synthase. Thus, we measured the total ATP production in the cells that were treated with AgNPs (1 μM), MS-275 (1 μM), and a combination of AgNPs (1 μM) and MS-275 (1 μM). For all tested samples, the level of ATP production was significantly affected. Remarkably, ATP production was dramatically reduced in cells treated with AgNPs and MS-275 (Figure 8C). Govender et al. [71] reported that AgNPs treatment increased mitochondrial depolarization significantly with an accompanying decrease in ATP concentration. AgNPs cause an imbalance of mitochondrial membrane potential that leads to mitochondrial damage, increases oxidative stress, reduces ATP content, damages DNA, and ultimately causes cell death in a variety of cancer cells [29,31,84]. Recently, Bao et al. [82] found that high concentrations of quisinostat, which is second generation of HDACi, significantly decreased intracellular ATP levels of A549 cells. Altogether, these data indicate that AgNPs and MS-275 can trigger mitochondria-mediated apoptosis by increasing ROS production and decreasing ATP generation in A549 cells.

### 2.9. AgNPs and MS-275 Activate Caspase 9 and 3 in A549 Cells

**Figure 9:**
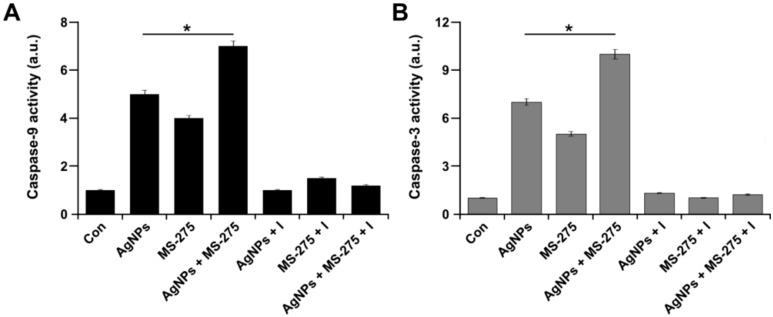
AgNPs and MS-275 induce caspase 9/3 activity in A549 cells. A549 cells were incubated with AgNPs (1 μM), MS-275 (1 μM), or a combination of AgNPs (1 μM) and MS-275 (1 μM) with and without a caspase-3/9 inhibitor for 24 h. The concentration of the *p*-nitroaniline released from the substrate was calculated from the absorbance values at 405 nm. The results represent the means of three separate experiments, and error bars represent the standard error of the mean. Treated groups showed statistically significant differences from the control group with Student’s *t*-test (* *p* < 0.05).

Mitochondrial alteration plays a major role in apoptosis. The alteration of MMP is responsible for the release of Ca^2+^ and cytochrome *c*, and activation of caspases resulted in cell death [85]. Caspases are the primary drivers and are able to cleave key intracellular substrates to promote cell death [86]. Caspases are categorized as initiator (caspase-2, -8, -9, and -10) or effector (caspase-3, -6, and -7) caspases, based on their position in apoptotic signaling cascades [86]. We chose to examine the activation of caspase 9 and 3, which are representative examples of initiators and effectors, respectively, and are also involved in the mitochondria-dependent intrinsic pathway. The intrinsic pathway is mainly regulated by mitochondria, which is not only the site where anti-apoptotic and pro-apoptotic proteins interact and determine cell fates, but also the origin of signals that initiate the activation of caspases through various mechanisms [87]. To determine the combinatorial effect of AgNPs and MS-275 on the activation of caspases-9 and -3, A549 cells were treated with AgNPs (1 μM), MS-275 (1 μM), or both AgNPs (1 μM) and MS-275 (1 μM) for 24 h, and then the activity of caspase was measured. The caspase activation was remarkably increased in the presence of AgNPs, MS-275, or the combination of both AgNPs and MS-275 (Figure 9A,B). The results from this experiment are consistent with an earlier report, suggesting that the combination of anticancer drugs, like gemcitabine and cisplatin, and HDAC inhibitors, like trichostatin A and tubostatin A, with AgNPs increases the activation of caspase-3 in a variety of cancer cells [6,7,34]. Several pro-apoptotic molecules, such as protease-activating factor and caspases, are released from the mitochondria during apoptosis in the presence of ATP. Our findings and those of Govender et al. [71] suggested that although ATP levels were reduced after AgNP treatment, the activity of caspase-9/3 was still elevated. Our findings are consistent with a previous report that demonstrated that the combination of resveratrol and genistein induced apoptosis by enhancing the activities of caspase-9 and caspase-3 in HeLa cells via lowered mitochondrial membrane potential [88]. MS-275 induces caspase-dependent apoptosis in B-cell chronic lymphocytic leukemia cells [89]. Similarly, Bao et al. [82] observed that in A549 cells exposed to quisinostat, the cleaved caspase-9 and caspase-3 protein levels were dramatically elevated. The results from this study suggest that the combination of AgNPs and MS-275 synergistically activate caspase-9 and 3 in A549 cells. The combined effect of AgNPs and MS-275 significantly activates caspase-9 and -3 more than single treatment. It was confirmed that AgNPs and MS-275 promote intrinsic apoptosis, which is a mitochondrion-centered cell death that is mediated by mitochondrial outer membrane permeabilization (MOMP), resulting in the activation of caspase-9 and subsequent activation of effector caspases [90]. An increase of ROS production, due to alteration of the mitochondria that consequently leads to loss of ΔΨm, seems to be a process of mitochondria-mediated early apoptosis [91].

### 2.10. Effect of AgNPs and MS-275 on Expression of Pro- and Anti-apoptotic Genes in A549 Cells

**Figure 10:**
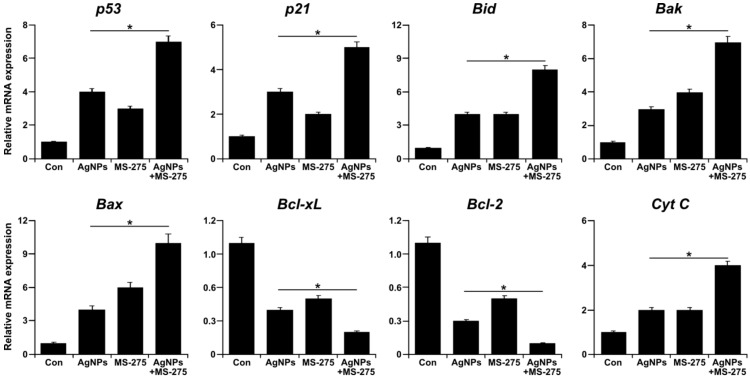
Effect of AgNPs and MS-275 on expression of apoptotic and anti-apoptotic genes in A549 cells. A549 cells were incubated with AgNPs (1 μM), MS-275 (1 μM), or a combination of AgNPs (1 μM) and MS-275 (1 μM) for 24 h. Relative mRNA expression was analyzed by qRT-PCR after the treatments. The results are expressed as the mean ± standard deviation of three independent experiments. The treated groups showed statistically significant differences from the control group by Student’s *t*-test. * *p* < 0.05.

The p53-mediated apoptosis is involved in inhibition of cell survival and anti-proliferative processes in response to different stress stimuli by directly activating apoptosis and promoting the release of bax [92], and is also involved in inducing a variety of caspase activity. Further, p53 interferes with mitochondrial integrity and function, leading to the release of pro-apoptotic molecules and the generation of ROS [31]. HDACi is known to activate p53 and as a consequence of activation of p53, a series of target genes, including the cyclin-dependent kinase inhibitor p21 resulting in cell cycle arrest and pro-apoptotic genes such as Bad, Bak, Bax, Puma, and Noxa, induce apoptosis [93]. To determine the effect of AgNPs and MS-275 on various pro- and anti-apoptotic gene expressions, A549 cells were treated with AgNPs (1 μM), MS-275 (1 μM), or both AgNPs (1 μM) and MS-275 (1 μM) for 24 h, and then expression analysis was measured by qRT-PCR. The results indicate pro-apoptotic genes, including p53, p21, Bid, Bax, Bak, and Cyt C, and down-regulating anti-apoptotic genes, such as Bcl-2 and Bcl-xL. The expression of pro-apoptotic genes showed a significant several-fold increase with the combination of AgNPs and MS-275 compared with single treatment. Conversely, the expression of anti-apoptotic genes showed a significant several-fold decrease with the combination of AgNPs and MS-275 compared with single treatment (Figure 10). In A549 cells exposed to quisinostat, the p53 signaling pathway was increased by expression of p53 and activation of p53 functions [82]. The HDACi-induced expression of p21 is dependent and independent of the p53 expression in cancer cells [94]. Sodium butyrate (NaB) HDACi is capable of alteration of the bcl-2 family protein expression and downregulates Bcl-xL, leading to apoptotic cell death in mesothelioma [95]. The HDACi-mediated intrinsic apoptotic pathway involves the interplay between pro- and anti-apoptotic Bcl-2 superfamily proteins [96]. The pro-apoptotic Bcl-2 members promote cytochrome *c* release, while anti-apoptotic Bcl-2 proteins such as Bcl-2, Bcl-xL, and Mcl-1 protect mitochondrial integrity [97,98]. The pro-apoptotic Bcl-2-family proteins Bax and Bak initiated mitochondrial membrane permeability to Bad, Bik, Bid, Bim, Bmf, Puma, and Noxa, which act as sensors of cellular stress for the activation of the intrinsic apoptotic pathway [99]. Among several anti-apoptotic members, Bcl-2 provides an acute protective function against apoptotic stimuli [100]. A significant decrease in Bcl-2 and Bcl-xL gene expression was associated with increased expression of Bid, Bak, Bax, and Cyt C, corroborated at the transcriptional level after AgNPs and MS-275 exposure. Bao et al. [82] found that A549 cells exposed to quisinostat resulted in a marked decrease in protein expressions of anti-apoptotic proteins Bcl-2 and Bcl-xl and a drastic increase of pro-apoptotic proteins Bax and Bim. Previously, we have demonstrated that AgNPs induce apoptosis through a p53-dependent pathway using the p53 inhibitor pifithrin α in human breast cancer cells [31,101]. Collectively, AgNPs and MS-275 induce cell death of tumor cells by apoptosis mainly through mitochondrial-mediated pathways by modulating Bcl-2 family proteins.

### 2.11. AgNPs and MS-275 Cause Apoptosis in A549 Cells

**Figure 11:**
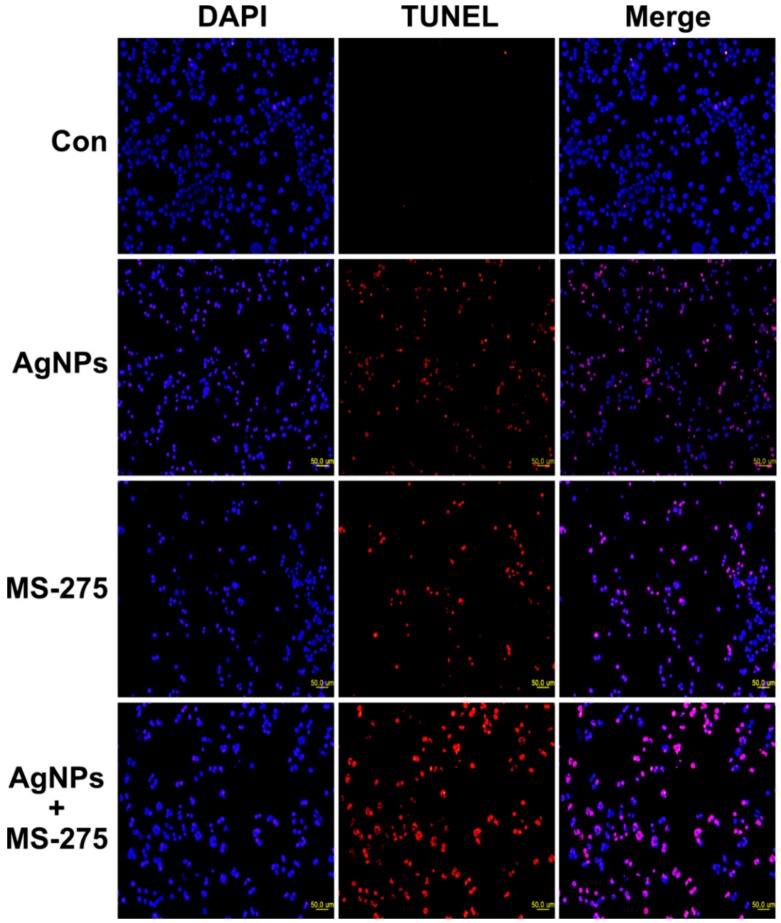
Analysis of apoptosis of A549 cells. A549 cells were incubated with AgNPs (1 μM), MS-275 (1 μM), or a combination of AgNPs (1 μM) and MS-275 (1 μM) for 24 h. After 24 h treatment, apoptosis was assessed by the transferase-mediated dUTP nick end labelling (TUNEL) assay; the nuclei were counterstained with DAPI. Representative images show apoptotic (fragmented) DNA (red staining) and the corresponding cell nuclei (blue staining). The images are ×50 μm magnification.

**Figure 12:**
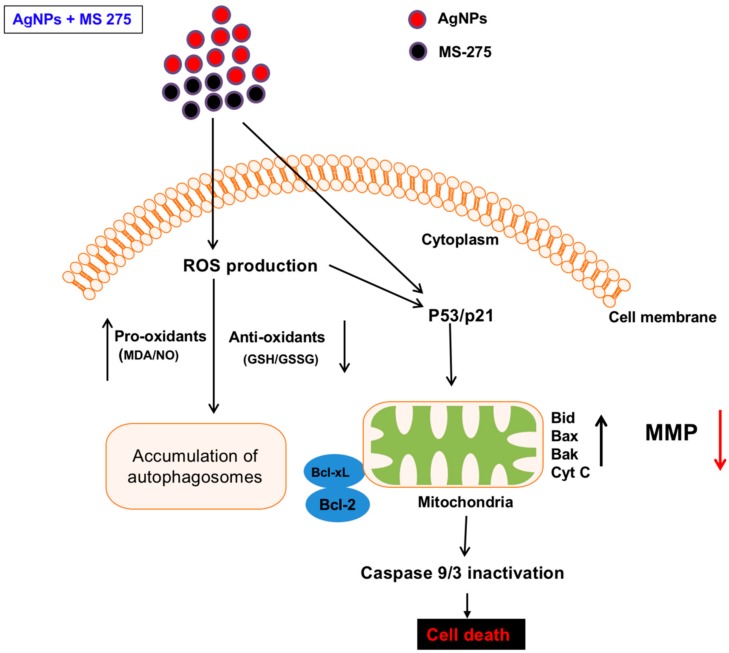
AgNPs and MS-275 induce cytotoxicity and apoptosis. Cell death is initiated by entry of AgNPs and MS-275 into the cells, which then triggers ROS production, LDH leakage, increase of pro-oxidants, and decrease of antioxidants, which leads to cytotoxicity. Altering of mitochondrial membrane potential (MMP) subsequently results in the release of pro-apoptotic mitochondrial proteins into the cytosol. This activates caspase-dependent processes culminating in cell death. Loss of MMP hampers mitochondrial function, which triggers a bio-energetic crisis due to loss of ATP. Depending on the intensity of the mitochondrial insult, the cell can undergo apoptosis, necrosis, and/or autophagic cell death, such as accumulation of autophagosomes. Therefore, combination of AgNPs and MS-275 causes significant cytotoxicity and apoptosis compared with single agent.

ROS are a byproduct of normal metabolism and have a significant role in cell signaling, such as cell survival and apoptosis. Excessive levels of ROS could generate oxidative stress, resulting in damage to key cellular components, including DNA, proteins, and lipids. Oxidation of DNA leads to the formation of lesions, including oxidized bases and DNA single- and/or double-strand breaks [102]. DNA damage induces an intra- and extracellular trigger signaling cascade responsible for biochemical and morphological features, including nuclear condensation, membrane blebbing, and DNA fragmentation, and eventually leads to cell death [103]. Therefore, we examined the effect of AgNPs and MS-275 on DNA-regulated apoptosis by deoxynucleotidyl transferase-mediated dUTP nick end labelling (TUNEL). DNA fragmentation analysis revealed that TUNEL positive cells were significantly higher in the combination of AgNPs and MS-275 compared with single treatment. Similarly, DNA fragmentation was remarkably increased compared with the control group (Figure 11). These results clearly indicate that excessive ROS and oxidative stress play an important role in AgNPs and MS-275 induced toxicity via DNA fragmentation. HDAC inhibitors, trichostatin A (TSA), sodium butyrate (NaB), and MS-275, induce cell death dose-dependently on the gastrointestinal NET cell lines CM and BON by DNA-fragmentation, resulting in an up to 12-fold increase of caspase-3 activation and downregulated Bcl-2 expression [54]. Several studies suggest that nanoparticles induce DNA damage and apoptosis in a variety of cancer cells, including human breast, ovarian, lung, cervical, and neuroblastoma cancer cells [6,7,30,31]. Similarly, vorinostat induces DNA double-strand breaks (DSBs) in normal (HFS) and cancer (LNCaP, A549) cells and causes an accumulation of ROS and caspase activation in certain transformed cells, but not in normal cells [104,105]. HDACi promote caspase/CAD dependent DNA fragmentation in glioma cells [50]. Altogether, our findings show that AgNPs and MS-275, which are more efficient for inducing apoptosis than a single agent, are effective killing adenocarcinoma cells by increasing DNA damage, thus leading to cell death. Hypothetical model demonstrates the combinatorial effect of AgNPs and MS-275 on cellular toxicity, apoptosis and autophagy (Figure 12). 

## 3. Materials and Methods

### 3.1. Materials 

Penicillin-streptomycin, trypsin-EDTA, RPMI 1640 medium, and 1% antibiotic-anti-mycotic were obtained from Life Technologies/Gibco (Grand Island, NY, USA). Wogonin, MS-275, fetal bovine serum (FBS), and the in vitro toxicology assay kit were purchased from Sigma-Aldrich (St. Louis, MO, USA). Silver nitrate, wogonin, and all other chemicals were purchased from Sigma-Aldrich unless otherwise stated. Human alveolar basal epithelial cells were obtained from ATCC (Manassas, VA, USA).

### 3.2. Synthesis and Characterization of AgNPs

The synthesis and characterization of AgNPs was performed using wogonin according to the method described previously [34]. 

### 3.3. Cell Viability Assays

Cell viability was measured using Cell Counting Kit-8 (CCK-8; CK04-01, Dojindo Laboratories, Kumamoto, Japan) according to manufacturer instructions.

### 3.4. Cell Proliferation Assay

Cell proliferation was evaluated according to the manufacturer’s instructions using BrdU Cell Proliferation Assay Kit and trypan blue exclusion assay was performed according to the method described earlier [106].

### 3.5. Cell Morphology

A549 cells were plated in six-well plates (2 × 10^5^ cells per well) and incubated with AgNPs (1 μM), MS-275 (1 µM), or a combination of AgNPs (1 μM) and MS-275 (1 µM) for 24 h. Cells cultured in medium without the addition of AgNPs or MS-275 were used as the control. 

### 3.6. Determination of Reactive Oxygen Species (ROS)

ROS were estimated according to a method described previously. The cells were seeded onto 24-well plates at a density of 5 × 10^4^ cells per well and cultured for 24 h. After washing twice with PBS, a fresh medium containing AgNPs (1 μM), MS-275 (1 µM), or a combination of AgNPs (1 μM) and MS-275 (1 µM), was then added to the cells for 24 h. The cells were then supplemented with 20 μM DCFH-DA, and the incubation continued for 30 min at 37 °C. 

### 3.7. Membrane Integrity

The membrane integrity of A549 cells was evaluated according to the manufacturer’s instructions (LDH Cytotoxicity Detection Kit; Takara, Tokyo, Japan). 

### 3.8. Measurement of TNF α

The secretion of TNF α was determined by measuring the culture supernatants using an ELISA kit from eBioscience (San Diego, CA, USA) according to the method described previously [58]. Briefly, the cells were treated with AgNPs (1 μM), MS-275 (1 µM), or a combination of AgNPs (1 μM) and MS-275 (1 µM) for 24 h and cell-free supernatant was transferred in triplicate into 96-well plates coated with capture antibody (Ab), and incubated overnight at 4 °C. The UV absorbance was measured at 450 nm with an ELISA reader (MolecularDevices, San Jose, CA, USA), and the concentration of cytokine (pg/mL) was calculated.

### 3.9. Assessment of Dead-Cell Protease Activity 

A dead-cell protease activity assay was performed according to the method described earlier [7]. The cytotoxicity was evaluated by treating the cells with AgNPs (1 μM), MS-275 (1 µM), or a combination of AgNPs (1 μM) and MS-275 (1 µM) for 24 h. The cytotoxicity was determined by association of intracellular proteases with a luminogenic peptide substrate (alanyl–alanylphenylalanyl–aminoluciferin). 

### 3.10. Measurement of ATP

The ATP level was measured according to the manufacturer’s instructions (Catalog Number MAK135) in A549 cells treated with AgNPs (1 μM), MS-275 (1 µM), or a combination of AgNPs (1 μM) and MS-275 (1 µM) for 24 h. The decreased levels of ATP and increased levels of ADP indicated the cytotoxicity of the treated cells. 

### 3.11. Determination of MDA, NO, GSH, and GSSG

The expression level of oxidative and anti-oxidative stress markers was measured as described previously [34]. MDA level was measured according to the method described earlier [34]. Briefly, the A549 cells were seeded into six-well microplates at 2.0 × 10^6^ cells per well. The cells were treated with AgNPs (1 μM), MS-275 (1 µM), or a combination of AgNPs (1 μM) and MS-275 (1 µM) for 24 h. After incubation, the cells were harvested and washed twice with an ice-cold PBS solution. The cells were collected and disrupted by ultrasonication for 5 min on ice. The cell extract (100 μL) was used to detect MDA according to the procedure recommended by the manufacturer of the pro- and antioxidant assay kit. 

### 3.12. Measurement of Mitochondrial Dysfunction 

MMP was measured as described previously using a cationic fluorescent indicator JC-1 (Molecular Probes, Eugene, OR, USA; [29]). Briefly, the cells were cultured in 75 cm^2^ culture flasks and exposed to AgNPs (1 μM), MS-275 (1 µM), or combination of AgNPs (1 μM) and MS-275 (1 µM) for 24 h. JC-1 is a lipophilic cation, which, in a reaction driven by ΔΨ_m_ in normal polarized mitochondria, assembles into a red fluorescence-emitting dimer, forming JC-1–AgNPs aggregates. 

A mitochondrial dysfunction analysis was carried out by determining the mitochondria copy number using real-time PCR amplification.

### 3.13. Transmission Electron Microscopy

The combination effect of MS-275 and AgNPs was performed by the cellular morphology of the treated cells. The cells were treated with AgNPs (1 μM), MS-275 (1 µM), or a combination of AgNPs (1 μM) and MS-275 (1 µM) for 24 h, following which the cells were harvested and fixed with a mixture of 2% paraformaldehyde and 2.5% glutaraldehyde in 0.2 M PBS for 8 h at pH 7.2. After fixation, the cells were incubated with 1% osmium tetroxide in PBS for 2 h. The stained sections on the grids were then examined with an H7000 TEM (Hitachi, Chiyoda-ku, Tokyo, Japan) at 80 kV.

### 3.14. Quantitative RT-PCR Analysis

According to the manufacturer’s instructions, total RNA was extracted from treated and untreated cells using the Dynabeads mRNA Direct Kit (ThermoFisher, Waltham, MA, USA). Real-time qRT-PCR was conducted using a Vill7 (Applied Biosystems, Foster City, CA, USA) and SYBR Green (Applied Biosystems, Foster City, CA, USA). Target gene expression levels were normalized to the *GAPDH* gene expression, which was unaffected byAgNPs (1 μM), MS-275 (1 µM), or a combination of AgNPs (1 μM) and MS-275 (1 µM). The real-time qRT-PCR primer sets are shown in Table 1. Real-time qRT-PCR was performed independently in triplicate, for each of the different samples, and the data are presented as the mean values of the gene expression levels measured in the treated samples versus the controls.

### 3.15. Measurement of Caspase 9/3 Activity

The caspase-3 activity was measured according to the method described previously [31]. The cells were treated with AgNPs (1 μM), MS-275 (1 µM), or a combination of AgNPs (1 μM) and MS-275 (1 µM) for 24 h, and then the activity of caspase-3/9 was measured in the cancer cells using a kit from Sigma-Aldrich Co., according to the manufacturer’s instructions. 

### 3.16. TUNEL Analysis

For the detection of apoptotic cells, A549 cells were treated with AgNPs (1 μM), MS-275 (1 µM), or a combination of AgNPs (1 μM) and MS-275 (1 µM) for 24 h, following which the terminal deoxynucleotidyl transferase-mediated dUTP nick end labelling (TUNEL) method was employed, using an in situ detection kit (Promega, Madison, WI, USA) according to the manufacturer’s instructions. 

### 3.17. Statistical Analysis

All assays were conducted in triplicate, and each experiment was repeated at least three times. The results are presented as means ± standard deviation. All the experimental data were compared using Student′s *t*-test. A *p*-value less than 0.05 was considered statistically significant.

## 4. Conclusions

Combination therapy, a treatment modality that combines AgNPs and MS-275, is a foundation of cancer therapy, which is more efficient and produces undesired side effects. To address this issue, we prepared AgNPs using wogonin as reducing and stabilizing agents via the green approach. The synthesized AgNPs combined with MS-275 show dose- and time-dependent inhibition of cell viability, cell proliferation, and alteration of cell morphology by increasing the oxidative stress. The combined treatment increases the cytotoxicity by ROS generation, LDH leakage, TNFα secretion, pro-oxidants, and decreasing the level of anti-oxidants. Moreover, the combined treatment potently induced apoptosis, which was accompanied by the alteration of mitochondrial membrane potential, mtDNA copy number, caspase activation, up and down regulation of Bcl-2 proteins, and DNA-fragmentation. The consolidation of AgNPs and HDACi enhances efficacy at low concentrations compared with either AgNPs or MS-275 alone. This approach could potentially reduce drug resistance. Therefore, these new strategies that target the survival pathways and provide efficient and effective results at an affordable cost are being considered. Our findings suggest that this combinatorial therapeutic approach is cost effective, easily accessible, and safe. In addition, this combined therapeutic approach is promising for mitigating tumor burden. We confirmed this observation and provided evidence of the ability of AgNPs to significantly potentiate the MS-275 effect, which is a viable and effective approach to use a minimal AgNPs concentration to achieve effective killing efficiency of cancer cells and to induce significant cytotoxic effects in cancer cells with HDACi.

## Figures and Tables

**Table 1:** Primers used for quantitative real-time PCR for the analysis of apoptotic and anti-apoptotic gene expression.

Gene	Primer
*Bax*	F: GAG AGG TCT TTT TCC GAG TGG
	R: GGA GGA AGT CCA ATG TCC AG
*p53*	F: AGG AAA TTT GCG TGT GGA GTA T
	R: TCC GTC CCA GTA GAT TAC CAC T
*Bak*	F: CTC AGA GTT CCA GAC CAT GTT G
	R: CAT GCT GGT AGA CGT GTA GGG
*Bcl-2*	F: CTG AGT ACC TGA ACC GGC A
	R: GAG AAA TCA AAC AGA GGC CG
*p21*	F: ATG TGG ACC TGT CAC TGT CTT G
	R: CTT CCT CTT GGA GAA GAT CAG C
*Cyt C*	F: GCGTGTCCTTGGACTTAGAG
	R: GGCGGCTGTGTAAGAGTATC
*Bid*	F: CCTTGCTCCGTGATGTCTTTC
	R: GTAGGTGCGTAGGTTCTGGT
*Bcl-xL*	F: GTAAACTGGGGTCGCATTGT
	R: CGATCCGACTCACCAATACC
*GAPDH*	F: AACGGATTTGGTCGTATTGGG
	R: TCGCTCCTGGAAGATGGTGAT
